# A Chronnectogram-ICA Framework for Tracking Continuously Evolving and Overlapping Functional Brain States

**DOI:** 10.64898/2026.10.14.759150

**Published:** 2026-10-16

**Authors:** Najme Soleimani, Sir-Lord Wiafe, Armin Iraji, Adrian Preda, Godfrey Pearlson, Tulay Adali, Vince Calhoun

## Abstract

The discrete-state model of brain dynamics has become the dominant paradigm in functional connectivity analysis, representing network activity as transitions among a fixed set of transiently recurring states while generally assuming that the states themselves do not evolve. We introduce a multilevel chronnectogram framework that relaxes this assumption and explicitly represents the higher-order temporal structure of brain dynamics, how distinct states couple and co-evolve over time, alongside the persistence of each state. The method integrates spatially guided independent component analysis (ICA) with subject-specific, state-guided dynamic ICA to capture evolving and overlapping connectivity patterns in a shared representational space. Validation on simulations with known ground truth shows recovery of continuous overlap and higher-order coupling structure invisible to discrete-state methods. Applied to resting-state functional magnetic resonance imaging from the FBIRN dataset (151 schizophrenia, 160 controls), the framework reveals that the same pair of brain states is shifted toward opposite extremes across patients, with strong synchrony in some and active anti-coupling in others, creating a redistribution of connectivity regimes invisible to conventional occupancy metrics, providing a substrate in which schizophrenia can appear both hyper- and hypo-volatile depending on the measure applied.

## Full Text Availability

The license terms selected by the author(s) for this preprint version do not permit archiving in PMC. The full text is available from the preprint server.

